# Lentivirus-mediated overexpression of OTULIN ameliorates microglia activation and neuroinflammation by depressing the activation of the NF-κB signaling pathway in cerebral ischemia/reperfusion rats

**DOI:** 10.1186/s12974-018-1117-5

**Published:** 2018-03-15

**Authors:** Hongbei Xu, Wenyi Qin, Xiao Hu, Song Mu, Jun Zhu, Wenhao Lu, Yong Luo

**Affiliations:** 1 Department of Neurology, the First Affiliated Hospital of Chongqing Medical University, Chongqing, 400016 China; 2 Laboratory Research Center, the First Affiliated Hospital of Chongqing Medical University, Chongqing, 400016 China; 3 Department of Integrated Chinese and Western Medicine, the First Affiliated Hospital of Chongqing Medical University, Chongqing, 400016 China; 4 Department of Neurology, Guizhou Provincial People’s hospital, Guizhou, 50002 China; 5 Department of Anus & Intestine surgery, the Affiliated Hospital of Guizhou Medical University, Guizhou, 550004 China

**Keywords:** OTULIN, Cerebral ischemia/reperfusion, Microglia, Neuroinflammation, NF-κB signaling pathway

## Abstract

**Background:**

Ischemic stroke-induced neuroinflammation is mainly mediated by microglial cells. The nuclear factor kappa B (NF-κB) pathway is the key transcriptional pathway that initiates inflammatory responses following cerebral ischemia. OTULIN, a critical negative regulator of the NF-κΒ signaling pathway, exerts robust effects on peripheral immune cell-mediated inflammation and is regarded as an essential mediator for repressing inflammation in vivo. The effect of OTULIN on inflammatory responses in the central nervous system (CNS) was previously unstudied. This current study investigated the anti-inflammatory effect of OTULIN both in vitro and in vivo in ischemic stroke models.

**Methods:**

Sprague-Dawley (SD) rats were subjected to transient middle cerebral artery occlusion (tMCAO) or an intraperitoneal injection of lipopolysaccharide (LPS). Overexpression of the OTULIN gene was utilized to observe the effect of OTULIN on ischemic stroke outcomes. The effect of OTULIN overexpression on microglia-mediated neuroinflammation was examined in rat primary microglia (PM) and in the microglial cell line N9 after induction by oxygen-glucose deprivation (OGD)-treated neuronal medium. The activation and inflammatory responses of microglia were detected using immunofluorescence, ELISA, and qRT-PCR. The details of molecular mechanism were assessed using Western blotting.

**Results:**

In the tMCAO rats, the focal cerebral ischemia/reperfusion injury induced a continuous increase in OTULIN expression within 72 h, and OTULIN expression was increased in activated microglial cells. OTULIN overexpression obviously decreased the cerebral infarct volume, improved the neurological function deficits, and reduced neuronal loss at 72 h after reperfusion, and it also inhibited the activation of microglia and attenuated the release of TNF-α, IL-1β, and IL-6 by suppressing the NF-κB pathway at 24 h after tMCAO. In vitro, OTULIN overexpression inhibited the microglia-mediated neuroinflammation by reducing the production of TNF-α, IL-1β, and IL-6 via depressing the NF-κB pathway in both PM and N9 cells.

**Conclusions:**

OTULIN provides a potential therapeutic target for ischemic brain injury by ameliorating the excessive activation of microglial cells and neuroinflammation through repressing the NF-κB signaling pathway.

**Electronic supplementary material:**

The online version of this article (10.1186/s12974-018-1117-5) contains supplementary material, which is available to authorized users.

## Background

Stroke is the second leading cause of morbidity, mortality, and long-term disability worldwide [1, 2]. Ischemic stroke, which is caused by a reduction in blood flow to the brain parenchyma, is the most common type of stroke [3]. The initial ischemic damage is followed by a wave of detrimental secondary events including oxidative stress, calcium and sodium overload, glutamate excitotoxicity, and inflammation. Neuroinflammation occupies a crucial role in the complicated pathologies that lead to ischemic brain injury and the subsequent reperfusion damage [4–6]. It is a dynamic process that mainly consists of aberrant activation of glial cells, particularly microglial cells, infiltration of circulating immune cells such as leukocytes, and subsequent production of inflammatory mediators [4–10].

Microglial cells are the principal resident immune cells in the central nervous system (CNS). Physiologically, resting microglial cells with small somas and threadlike processes continuously monitor the CNS microenvironment [11–14]. Once cerebral ischemia occurs, microglial cells undergo a series of phenotypic changes, such as morphological transformation, proliferation, migration, phagocytosis, and secretion of inflammatory mediators [15–17]. Upon activation, microglia retract their processes and transform into a cellular morphology possessing few and thicker processes or an amoeboid shape [18]. Activated microglial cells can act as double-edged swords in ischemic stroke. On the one hand, activated microglial cells migrate to the ischemic area to clear the harmful agents and maintain tissue homeostasis [19]. On the other hand, over-activated microglia are detrimental and cause uncontrolled inflammation by producing excessive inflammatory cytokines, chemokines, and oxygen/nitrogen free radicals, such as nitric oxide (NO), tumor necrosis factor-α (TNF-α), interleukin-1β (IL-1β), interleukin-6 (IL-6), and reactive oxygen species (ROS), which exacerbate tissue damage and neuronal death [20–22]. Inhibiting the over-activation of microglia and inflammatory responses in the early stage of acute ischemic stroke can efficiently prevent brain damage and, therefore, improve neurological outcome [23–25].

The canonical transcriptional factor nuclear factor kappa B (NF-κB) is the key transcriptional factor widely known to be associated with the activation of microglia and the subsequent inflammatory responses following cerebral ischemia [26, 27]. NF-κB mainly exists as a heterodimer composed of p65 and p50. In resting cells, NF-κB resides in the cytoplasm and is bound to inhibitory IκB proteins (with NF-κB inhibitor protein alpha (IκBα) playing a leading role). After various stimuli, IκBα is phosphorylated and degraded by IκB kinases (IKKs), and that degradation leads to the nuclear translocation of p65/p50 NF-κB and promotes transcription of many pro-inflammatory cytokine genes [28, 29]. IKKs mainly consist of three subunits, including the kinases IKKα and IKKβ and the regulatory subunit IKKγ (NEMO) [30]. The activation of IKKs is a critical step in NF-κB activation [31]. Proper inhibition of NF-κB p65 nuclear translocation can effectively inhibit the activation of microglia and neuroinflammation following ischemic stroke [32–35].

OTULIN (also termed gumby/FAM105B), a new deubiquitinase discovered in 2013, has been well demonstrated to be a critical negative regulator of the canonical NF-κΒ pathway [36–39]. OTULIN was reported to be involved in NF-κΒ-dependent inflammatory signaling and was regarded as an essential endogenous regulator for controlling inflammatory responses [36, 40–42]. Recessively inherited loss-of-function mutations in OTULIN caused patients to have an early onset severe autoinflammatory disease termed otulipenia/ORAS (OTULIN-related autoinflammatory syndrome) [40, 42]. Moreover, OTULIN exerts robust effects on inflammation mediated by peripheral immune cells. Tamoxifen-induced OTULIN deficiency in immune cells led to an acute, serious multi-organ inflammatory phenotype [40]. However, the role of OTULIN in the CNS remained unknown. Therefore, the present study mainly investigated whether OTULIN affects the activation of microglial cells and neuroinflammation via regulating the NF-κB signaling pathway in focal cerebral ischemia/reperfusion rats.

## Methods

### Animals

Adult male Sprague-Dawley (SD) rats (250–300 g) and neonatal (1 day old) SD rats were purchased from the Experimental Animal Center of the Chongqing Medical University. The adult animals were housed in a 12-h light/dark cycle condition with a temperature of 22 ± 2 °C and humidity of 65 ± 5%. The experimental protocol applied in the present study was approved by the Ethics Committee for Animal Experimentation of the First Affiliated Hospital of Chongqing Medical University. All procedures conducted were in accordance with the guidelines of the National Institutes for Animal Research.

### Animal surgery for the focal cerebral ischemia/reperfusion model

A focal cerebral ischemia/reperfusion model was established by transient middle cerebral artery occlusion (tMCAO) as previously described [43, 44]. Briefly, adult SD rats were anesthetized with 3.5% chloral hydrate (1 ml/100 g) by intraperitoneal (IP) injection. During surgery, the core body temperature was continuously detected by a rectal probe and maintained at 38 ± 0.5 °C by a thermostatically controlled infrared lamp (FHC, Bowdoinham, ME, USA). A ventral midline neck incision was made to expose the right common carotid artery (CCA), the right external carotid artery (ECA), and the right internal carotid artery (ICA). Branches of the ECA were then ligated and cut off at 2.0 mm from the bifurcation of the CCA, and a heparin-dampened monofilament nylon suture (Ethicon Nylon Suture; Ethicon Inc., Osaka, Japan) with a rounded tip was gently inserted 18–20 mm from an incision in the ECA to occlude the right middle cerebral artery (MCA), confirmed with mild resistance. After 2 h of occlusion, the filament was withdrawn to restore blood flow. Successful occlusion was confirmed by a decrease in the regional cerebral blood flow to 20% and recovered to more than 80% of the baseline as detected by a laser-Doppler flowmeter (PeriFlux 5000, Perimed AB, Sweden). The animals that did not meet this requirement were excluded from the experiment. The Sham-operated rats underwent all the same surgical procedures except that the monofilament was not inserted to the MCA origin. All animals were closely monitored until they recovered from anesthesia, after which they were returned to their cages.

### Lipopolysaccharide administration

To induce a pure brain inflammation, lipopolysaccharide (LPS) (Sigma-Aldrich, St Louis, MO, USA, 0111: B4) was diluted with sterile PBS and administered at a dose of 500 μg/kg (IP). This dose of LPS is sufficient to cause brain inflammation without causing neuron death and has no effect on motor activity [45–47]. The control group received the same volume of sterile saline. At 24 h after injection, the rats were sacrificed for later experiments. LPS-induced neuroinflammation models were successful, as shown by sharp increases in TNF-α, IL-1β, and IL-6 mRNA levels detected by real-time quantitative reverse transcription polymerase chain reaction (qRT-PCR; data not shown).

### Primary cortical neuron cultures and oxygen-glucose deprivation

Primary cortical neurons were cultured with a modified method reported previously [48]. The meninges and blood vessels were isolated from the cerebral cortices of 1-day-old SD rats. The tissues were chopped mechanically, digested with 0.25% trypsin at 37 °C for 30 min, neutralized with 10% fetal bovine serum (FBS; Gibco Co., USA), and triturated with a Pasteur pipette. Then, the cells were seeded on poly-d-lysine pre-coated six-well plates (1.5 × 10^6^ cells per well) and cultured in Dulbecco’s modified Eagle’s medium/F12 medium (DMEM/F12; Gibco Co., USA) containing 10% FBS in a humidified chamber with 5% CO_2_ at 37 °C for 5 h. The medium was replaced with Neurobasal medium (Gibco Co., USA) supplemented with 2% B27 and 0.5 mM glutamine. Half of the culture medium was replaced every 3 days. Arabinosylcytosine (sc-201628, Santa Cruz Co., USA) was added (5 μg/ml) at 3 days after incubation to inhibit the growth of non-neuronal cells. Cell immunofluorescence results showed that the cultured cells stained with NeuN (neuronal nuclei) exceeded 90% (data not shown). Neurons were identified by staining with anti-NeuN (MAB377, Millipore Co., Germany, 1:200).

At 7 days after culture, neurons were subjected to oxygen-glucose deprivation (OGD) according to a previously described method with some modifications [49]. Briefly, the neuronal culture medium was replaced with glucose-free D-Hanks solution (HyClone, USA). Next, the neurons were maintained in an anaerobic chamber filled with 94% N_2_, 1% O_2_, and 5% CO_2_ for 3 h. For re-oxygenation, the cells were removed from the anaerobic chamber (Thermo 3111; Thermo Fisher Scientific Inc., USA) and returned to normal oxygen, and the D-Hanks solution was subsequently replaced with Neurobasal medium. After 24 h of re-oxygenation, the OGD-treated conditioned medium was collected for later use. The supernatant without OGD treatment served as a negative control.

### Primary microglial and N9 microglial cell cultures

A primary microglial culture was prepared following a method previously described with some modifications [50]. Briefly, the meninges and blood vessels in the rat cortex were removed from SD rats on postnatal day 1. Cortical tissue was dissociated with 0.25% trypsin at 37 °C for 30 min, and the cell suspension was filtered via a 70-μm filter. After centrifugation at 1000 rpm for 10 min, the cells were re-suspended by Pasteur pipette and then grown in flasks in a humidified atmosphere containing 5% CO_2._ The culture medium, DMEM/F12 supplemented with 10% FBS, was refreshed every 3 days. After 2 weeks, microglial cells were isolated from the mixed glial cells by shaking the flasks on a rotary table concentrator at 200 rpm for 4 h at 37 °C. The purity of the cultured microglia was more than 95%, as detected by ionized calcium-binding adaptor molecule 1 (Iba-1; NB100-1028, Novus Co., USA, 1:200) immunocytochemical staining (data not shown). Iba-1 is a general marker for microglial cells.

N9 microglial cells (kindly provided by the Department of Anesthesia, the Affiliated Children’s Hospital of Chongqing Medical University) were cultured in DMEM-F12 supplemented with 10% FBS, 100 U/ml penicillin, and 100 U/ml streptomycin at 37 °C in a humidified environment containing 5% CO_2_.

### Lentivirus construction, intracerebroventricular administration, and cell transfection

The lentivirus for overexpressing OTULIN (LV-OTULIN) and the control lentivirus (LV-Scramble) were obtained commercially from GenePharma Corporation (Shanghai, China).

Seven days before tMCAO surgery or LPS treatment, rats received an intracerebroventricular (ICV) injection of LV-OTULIN or LV-Scramble. The ICV injection was performed as previously described [51]. Briefly, rats were anesthetized with 3.5% chloral hydrate (1 ml/100 g) and placed in a stereotaxic apparatus (Stoelting, USA). A cranial hole on the right hemisphere was drilled 1.3 mm lateral and 1.5 mm posterior to bregma. A 10-μl Hamilton syringe (Hamilton Co., Reno, NV, USA) was stereotaxically inserted into the hole prepared for the ICV injection (3.8 mm under the dural surface). Five microliters of LV-OTULIN (1 × 10^9^ transduction units (TU)/ml) or LV-Scramble (1 × 10^9^ TU/ml) was injected into the right hemisphere at a rate of 0.5 μl/min. The needle was kept in place for an additional 5 min to prevent reflux and was then removed slowly. The expression of TREM2 mRNA and protein was significantly upregulated by OTULIN overexpression before the induction of tMCAO (Additional file 1: Figure S1).

Primary microglia and N9 microglial cells were seeded in six-well plates and transfected continuously with LV-OTULIN or LV-Scramble for 72 h. Subsequently, the medium was replaced with fresh medium. The effects of gene interference on OTULIN expression both in vivo and in vitro were verified using qRT-PCR and Western blotting.

### Induction of inflammation in primary microglia and N9 cells with OGD-conditioned media

To mimic the in vivo ischemia model, DMEM/F12 medium from primary microglia and N9 cells was replaced with OGD-treated conditioned medium (OGD[+]CM), and OGD-untreated conditioned medium (OGD[−]CM)-treated groups served as control groups.

### Neurobehavioral assessment

Neurobehavioral assessment was performed at 72 h after reperfusion, based on the Longa score [43], modified Bederson score [52], and modified Garcia score [53] determined by an examiner blinded to the experimental conditions of the animals.

The Longa score was used to detect motor functions. It was graded as follows: 0, no observable deficits; 1, failure to fully extend the left forepaw; 2, difficulty in extending the left forelimb and circling to the left; 3, failing to the left side; and 4, no spontaneous walking and decreased level of consciousness. The higher the score is, the more severe the damage. Animals that scored 2 and 3 were included in this study, and the tMCAO rats that scored 0, 1, or 4 were excluded.

The Bederson score was applied to evaluate global neurological function. It was graded on a 5-point scale: 0, no observable deficits; 1, lost forelimb flexion; 2, lost forelimb flexion with lower resistance to lateral push; 3, unidirectional circling; 4, longitudinal spinning or seizure activity; and 5, no movement. The higher the score is, the more severe the damage.

The Garcia score was used to evaluate the sensorimotor function as follows: symmetry of limbs (0–3 points), spontaneous activity (0–3 points), forepaw outstretching (0–3 points), climbing (1–3 points), body proprioception (1–3 points), and response to vibrissal touch (1–3 points). The lower the score is, the more severe the damage.

### Measurement of cerebral infarct volume

The infarct volume was determined by 2,3,5-triphenyltetrazolium chloride (TTC; Sigma-Aldrich, USA) staining according to a previous method [54]. Briefly, rats were euthanized and decapitated immediately at 72 h after reperfusion. The brains were rapidly removed and frozen for 20 min at − 20 °C. Then, the brains were coronally sectioned into five consecutive slices (2 mm thick), stained with 2% TTC at 37 °C for 10 min, and then fixed with 10% formaldehyde. The slices were photographed with a digital camera (Canon IXUS, Canon Co., Japan), and image analysis software (Image-Pro Plus 6.0, Media Cybernetics Co. USA) was used to calculate the infarct volume via the following formula: percentage hemisphere lesion volume (% HLV = {[total infarct volume−(right hemisphere volume−left hemisphere volume)]/left hemisphere volume} × 100%). The measurements were performed in a blind manner.

### Western blot analysis

**Fig. 1:**
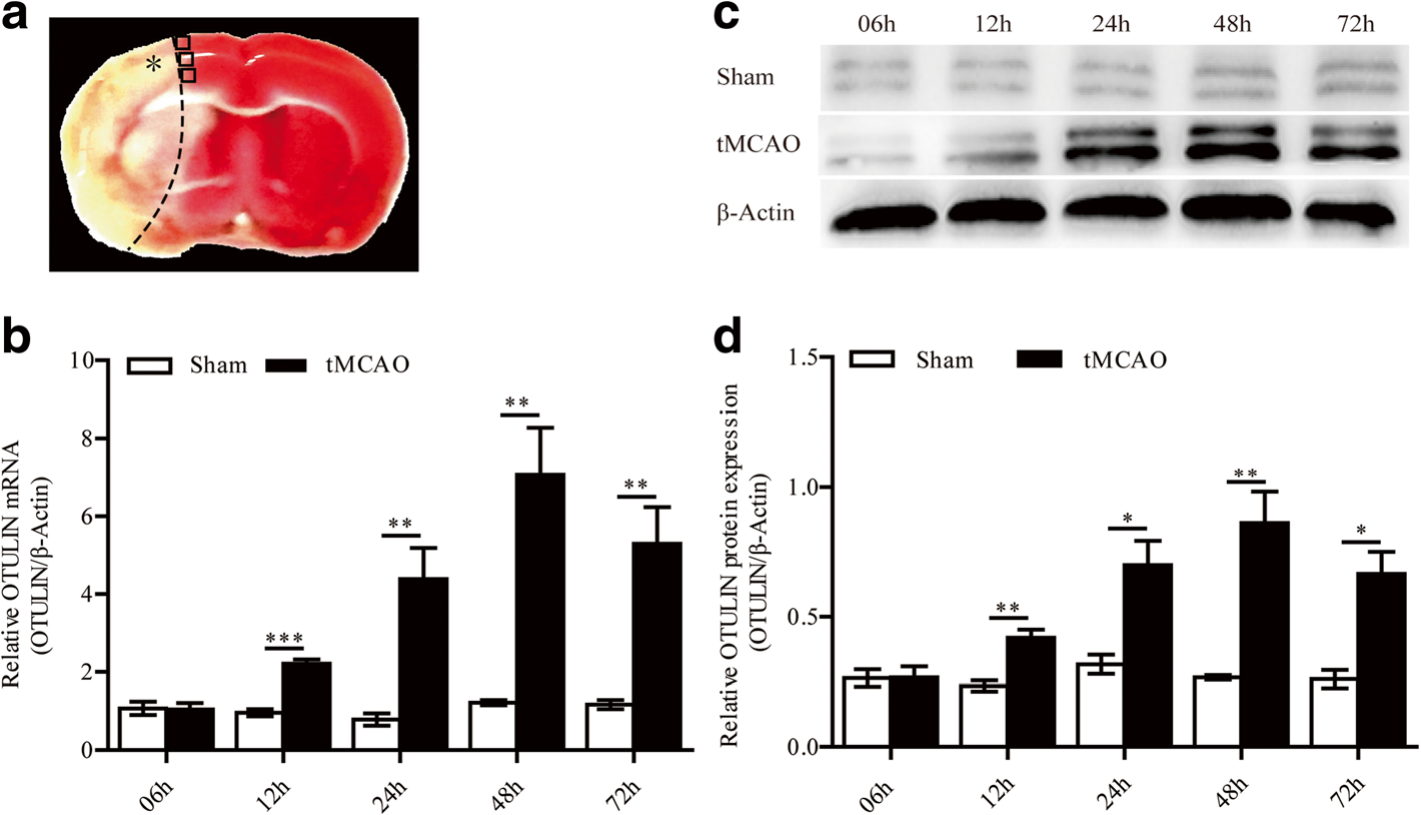
Cerebral ischemia/reperfusion increased the levels of OTULIN mRNA and protein at the indicated time points (6, 12, 24, 48, and 72 h) after reperfusion following 2 h of ischemia. **a** A coronal brain section (stained with TTC) is labeled with black boxes to show the regions analyzed (asterisk represents ischemic core area; boxes represent the ischemic penumbra proximal to the ischemic core area). **b** OTULIN mRNA levels in the Sham and tMCAO groups at each time point were detected by qRT-PCR (*n* = 5). **c** OTULIN protein levels in the Sham and tMCAO groups at each time point were examined with Western blot. **d** The histogram presents the quantitative analysis of OTULIN protein levels at the indicated time points in the Sham and tMCAO groups (*n* = 3). All values are presented as the means ± SEMs; ***P < 0.001, **P < 0.01, and *P < 0.05 versus the Sham group

The brain tissues in the ischemic penumbra (the location is illustrated in Fig. 1a) were removed at indicated times. The cultured cells were digested with 0.25% trypsin and then centrifuged at 1500 rpm for 15 min. The samples were homogenized in radioimmunoprecipitation assay (RIPA) lysis buffer (no. P0013B, Beyotime, Shanghai, China) supplemented with phenylmethane sulfonyl fluoride (PMSF; Beyotime, Shanghai, China) and additional phosphatase inhibitors to detect phosphorylated proteins. Total protein was extracted from the supernatants after centrifugation at 12,000 rpm for 15 min. Nuclear and cytoplasmic protein fractions were extracted by using a Nuclear and Cytoplasmic Protein Extraction Kit (no. AR0106, Boster, Beijing, China) according to the manufacturer’s instructions. Concentrations of the protein extracts were detected by using a BCA Protein Assay Reagent Kit (Beyotime, Shanghai, China). Total protein (50 μg) was separated by SDS-PAGE and transferred to polyvinylidene fluoride (PVDF) membranes (Millipore Co., USA). The membranes were blocked with 5% non-fat milk for 1 h at room temperature and incubated at 4 °C overnight with the following primary antibodies: anti-OTULIN polyclonal rabbit antibody (no. 14127, Cell Signaling Technology, USA, 1:1000), anti-NF-κB/p65 rat monoclonal antibody (no. 8242, Cell Signaling Technology, USA, 1:1000), anti-IκBα rabbit monoclonal antibody (no. 4812, Cell Signaling Technology, USA, 1:1000), anti-phospho-IκBα (Ser32) rabbit monoclonal antibody (no. 2859, Cell Signaling Technology, USA, 1:500), and anti-β-actin rabbit monoclonal antibody (no. 4970, Cell Signaling Technology, USA, 1:1000). After washing three times with TBST, the membranes were incubated with a specific horseradish peroxidase-conjugated secondary antibody for 1 h at 37 °C. A gel imaging instrument (Vilber Lourmat fusion FX 7 Spectra, France) and analysis software (FUSION-CAPT, France) were used to scan the immunoblots and analyze the relative density of each band. The final results are presented as the ratios of the optical densities of targeted proteins to those of β-actin.

### Real-time quantitative reverse transcription polymerase chain reaction analysis

Total RNA of brain tissue or cultured cells was extracted by using Trizol (Takara Biotechnology, Japan) and converted to cDNA by using a Prime Script TM RT Reagent kit (Takara Biotechnology, Japan). The real-time quantitative reverse transcription polymerase chain reaction (qRT-PCR) was performed on a Q5 Gradient Real-Time PCR Detection System (Bio-Rad Co., USA) with the SYBR Green System (SYBR Premix Ex TaqTMII, Takara Co., Japan). Cycle conditions included heating for 30 s at 95 °C, followed by 40 cycles of 5 s at 95 °C, 30 s at 60 °C, and 60 s at 72 °C. The melting curve of each gene was performed to ensure specific amplification. All gene-specific expression values were normalized against β-actin gene expression in the same samples. All primer sequences are shown in Table 1.

**Table 1:** List of primers used for qRT-PCR

Gene	Sequence	
OTULIN	Forward	5′-TGTGGCTCCTGAAATGGATATTATG-3′
	Reverse	5′-CTCTGACAGGGATGTTATAGTGCCG-3′
β-Actin	Forward	5′-TGTCACCAACTGGGACGATA-3′
	Reverse	5′- GGGGTGTTGAAGGTCTCAAA-3

### Enzyme-linked immunosorbent assay

The levels of TNF-α, IL-1β, and IL-6 in brain homogenate and cell supernatants were measured by using enzyme-linked immunosorbent assay (ELISA) kits (no. EK0526 96T, EK0393, and EK0412 96T, BOSTER Co., China) following the manufacturer’s instructions.

### Immunofluorescence

The slides of brain sections were fixed with 4% formaldehyde solution for 30 min at room temperature, incubated with 1% Triton X-100 for 30 min, and blocked with 5% goat or donkey serum for 1 h at 37 °C. Subsequently, sections were incubated overnight at 4 °C with the following primary antibodies: OTULIN (bs-14689R, Bioss Co., Beijing, China, 1:50), NeuN (MAB377, Millipore Co., Germany, 1:200), MAP2 (4542, Cell Signaling Technology, USA, 1:100) or Iba-1 (NB100-1028, Novus Co., USA, 1:200). After washing three times with PBS, sections were reacted with the following fluorescent secondary antibodies at 37 °C for 1 h: Alexa Fluor 594-conjugated goat anti-rabbit IgG (H+L; SA00006-4, Proteintech, 1:200), Alexa Fluor 488-conjugated goat anti-mouse IgG (H+L; SA00006-1, Proteintech, 1:200), FITC-conjugated AffiniPure donkey anti-goat IgG (H+L; SA00003-3, Proteintech, 1:200), and 594-conjugated AffiniPure donkey anti-rabbit IgG (H+L; SA00006-8, Proteintech, 1:200). DAPI (Sigma, USA, 1:200) was used to stain cellular nuclei at 37 °C for 10 min. All images were observed and acquired using an A1+R laser confocal microscope (Nikon, Tokyo, Japan).

### Statistical analyses

The Longa score and Bederson score data were analyzed using Kruskal-Wallis tests followed by post hoc Dunn’s multiple comparison tests. Unpaired Student’s *t* tests were used when two groups were compared. All other quantitative data were analyzed using one-way ANOVA followed by Tukey’s post hoc test for multiple comparisons. Statistical analyses were performed using SPSS 19.0. All values are expressed as the means ± SEMs, and values of *P* < 0.05 were considered statistically significant.

## Results

### Cerebral ischemia/reperfusion injury increased OTULIN expression in rats

To analyze the time course of OTULIN expression following cerebral ischemia/reperfusion, we detected the levels of OTULIN mRNA and protein in the ischemic penumbra of the cerebral cortex (Fig. 1a) within 72 h after reperfusion by Western blot and qRT-PCR. The rats were randomly divided into the Sham group and the tMCAO group. OTULIN mRNA expression in the tMCAO group increased gradually with the prolongation of reperfusion time and peaked at 48 h, followed by a decrease at 72 h. Moreover, the levels of OTULIN mRNA at indicated times in the tMCAO group remained significantly higher than those in the Sham group except at 6 h (Fig. 1b, *n* **=** 5 per group for RT-qPCR).

Consistent with the qRT-PCR results, the Western blot data indicated that OTULIN protein levels in the tMCAO group were markedly increased compared with those in the Sham group at each time point except at 6 h (Fig. 1c, d, *n* **=** 3 per group for Western blot). Together, these results suggest that cerebral ischemia induced an endogenous increase in OTULIN expression in the ischemic penumbra of the cerebral cortex.

### OTULIN overexpression protected against ischemic injury in focal cerebral ischemia/reperfusion rats

**Fig. 2:**
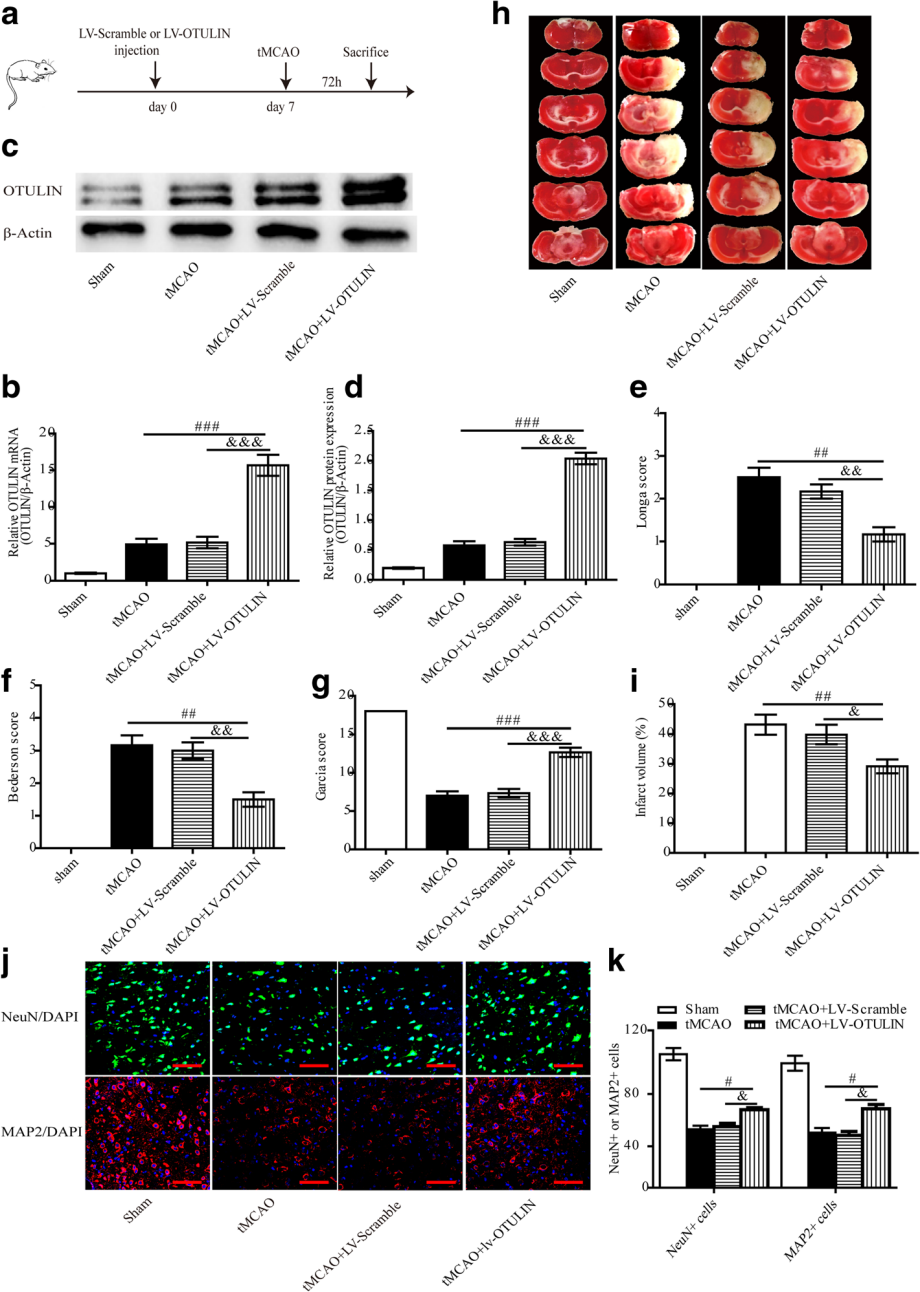
Lentivirus-mediated OTULIN overexpression exerted a neuroprotective role at 72 h after reperfusion in focal cerebral ischemia/reperfusion rats. **a** Schematic representation of the animal experiments over time. OTULIN mRNA (**b**, *n* = 5) and protein (**c** and **d**, *n* = 3) levels were elevated effectively by LV-OTULIN. The Longa score (**e**, *n* = 6), Bederson core (**f**, *n* = 6), and Garcia score (**g**, *n* = 6) were applied to assess neurological function deficits. **i** Quantification of infarction volumes was calculated based on TTC staining (**h**, *n* = 5**)**. MAP2 (**j**, *n* = 6) and NeuN (**j**, *n* = 6) antibodies were used to stain neurons in the ischemic penumbra; scale bar = 75 μm. Quantification of the number of MAP2^+^/NeuN^+^ neurons was presented in panel **k**. All values are presented as the means ± SEMs; ###*P* < 0.001, ##*P* < 0.01, and #*P* < 0.05 versus the tMCAO group; &&&*P* < 0.001, &&*P* < 0.01, and &*P* < 0.05 versus the tMCAO+LV-Scramble group

To investigate the effect of OTULIN on stroke outcomes, cerebral infarct volume, neurobehavioral assessments, and neuronal loss in each group were detected at 72 h after reperfusion. OTULIN expression was enhanced by ICV injection of LV-OTULIN, and an empty vector (LV-Scramble) was injected as a control (Fig. 2a). The tMCAO model was established at 7 days after ICV injection, and 72 h later, animals were sacrificed for subsequent experiments (Fig. 2a). The rats were divided into four groups: Sham, tMCAO, tMCAO+LV-Scramble, and tMCAO+LV-OTULIN. As expected, the LV-OTULIN lentivirus effectively promoted OTULIN mRNA and protein expression as determined by qRT-PCR (Fig. 2b, *n* = 5 per group) and Western blot (Fig. 2c, d, *n* = 3 per group) compared to those in the tMCAO group and the tMCAO+LV-Scramble group.

The Longa score, Bederson score, and Garcia score consistently showed that the tMCAO group exhibited obvious neurological dysfunction compared with the Sham group. The neurological function deficits in the tMCAO+LV-OTULIN group were obviously improved compared to those in the tMCAO group and the tMCAO+LV-Scramble group in terms of the Longa score (Fig. 2e, *n* **=** 6 per group), Bederson score (Fig. 2f, *n* **=** 6 per group), and Garcia score (Fig. 2g, *n* = 6 per group). Moreover, rats in the tMCAO+LV-OTULIN group displayed significantly smaller infarct volume in the cortex and striatum compared to those in the tMCAO group or tMCAO+LV-Scramble group (Fig. 2h, i, *n* = 5 per group). The infarct volume in the tMCAO group was similar to that in the tMCAO+LV-Scramble group **(***P* > 0.05; Fig. 2h, i**)**. We further examined NeuN- and MAP2-stained neurons to assess the brain infarct damage. The qualitative analysis revealed that OTULIN overexpression markedly increased both NeuN-positive (Fig. 2j, k, *n* = 6 per group) and MAP2-positive (Fig. 2j, k, *n* = 6 per group) cells in the ischemic penumbra compared with that in the tMCAO group or the tMCAO+LV-Scramble group at 72 h following reperfusion. Collectively, these data indicate that the outcome of ischemic stroke could be improved by OTULIN overexpression.

### OTULIN overexpression attenuated microglia activation in focal ischemia/reperfusion rats

**Fig. 3:**
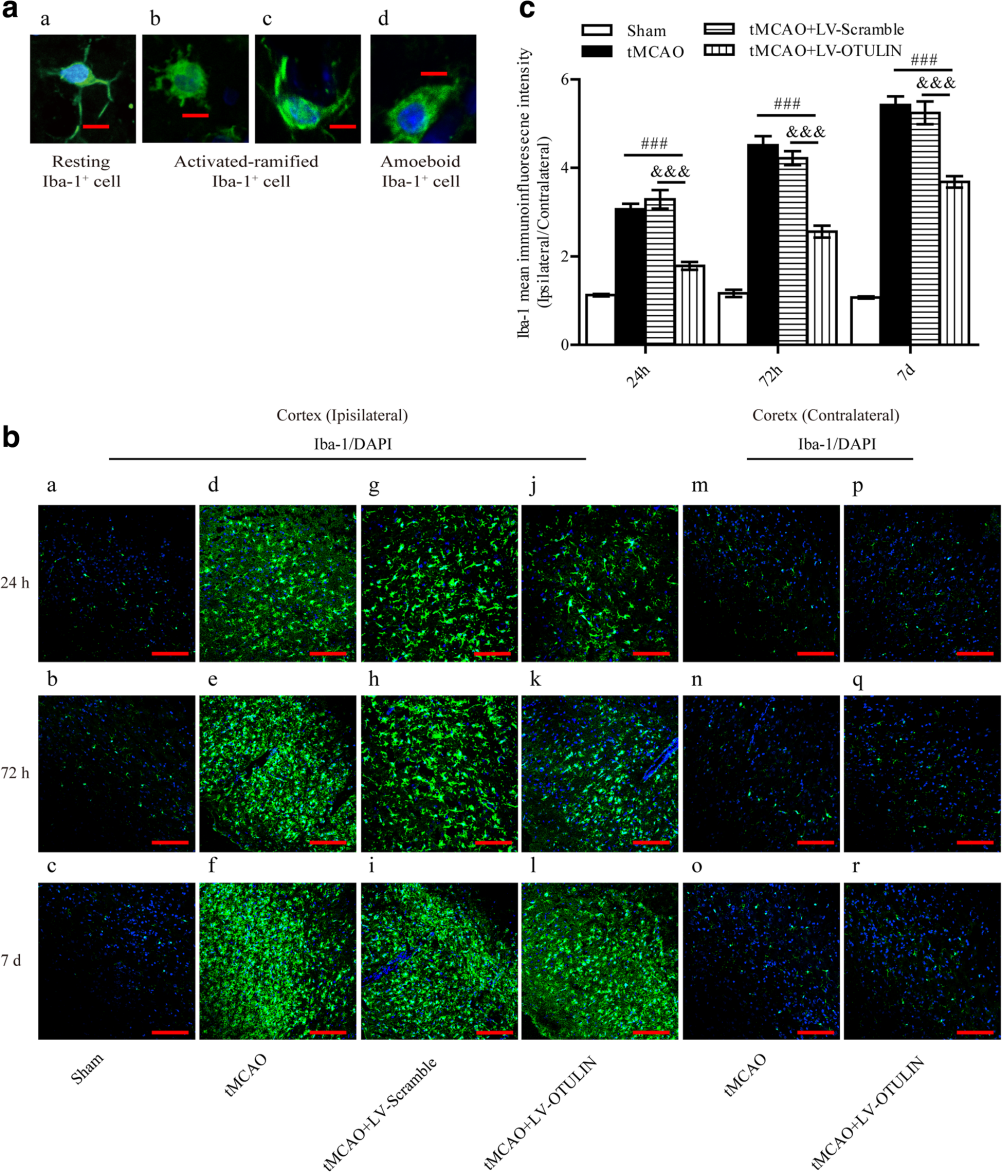
OTULIN overexpression reduced the number of Iba-1^+^ microglial cells in the cortical ischemic penumbra in focal cerebral ischemia/reperfusion rats. **A** Representative images of Iba-1^+^ microglial cells that were morphologically classified into three types; scale bar = 10 μm. **B** Iba-1^+^ microglial cells in the ischemic penumbra and contralateral cortex of each group were detected by confocal immunofluorescence at 24 h, 72 h, and 7 days; scale bar = 75 μm. **C** The histogram represents the quantitative analysis of mean Iba-1 immunofluorescence intensity in each group (*n* = 6). Data are presented as the means ± SEMs; ###*P* < 0.001 versus the tMCAO group; &&&*P* < 0.001 versus the tMCAO+LV-Scramble group

To investigate the effect of OTULIN overexpression on microglia activation, we analyzed the immunoreactivity of Iba-1^+^ cells in the cortical ischemic penumbra. The rats were divided into four groups including: Sham, tMCAO, tMCAO+LV-Scramble, and tMCAO+LV-OTULIN (*n* = 6 per group). Morphological change is one of the classic features of microglia activation after ischemic stroke [55]. Iba-1^+^ cells were mainly classified into three types according to a common morphological classification: resting cells, activated-ramified cells, and amoeboid cells. Resting Iba-1^+^ cells featured small cell bodies and thin-branched processes (Fig. 3A(a)). Activated-ramified Iba-1^+^ cells (Fig. 3A(b), (c)) also had processes, but their cell bodies and processes were thick. Amoeboid Iba-1^+^ cells (Fig. 3A(d)) were characterized by enlarged cell bodies without processes. In the Sham group, almost all microglia remained in the resting state at 24 h, 72 h, and 7 days. At 24 h after stroke, Iba-1^+^ microglia mainly became activated-ramified cells, and a few amoeboid Iba-1^+^ cells in the penumbra were observed in all groups. The mean Iba-1 immunofluorescence intensity in the tMCAO+LV-OTULIN group was obviously more decreased than that in the tMCAO group or tMCAO+LV-Scramble group (Fig. 3B(d, g, j), C). At 72 h after reperfusion, amoeboid Iba-1^+^ cells obviously appeared and accumulated in the penumbra. The mean Iba-1 immunofluorescence intensity in the tMCAO+LV-OTULIN group (Fig. 3B(k), C) was significantly lower than that in the tMCAO group (Fig. 3B(e)) or the tMCAO+LV-Scramble group (Fig. 3B(h)). Almost all Iba-1^+^ activated cells become amoeboid in shape, and more prominent microglia accumulated in the penumbra at 7 days after reperfusion; moreover, the mean Iba-1 immunofluorescence intensity was more obviously decreased in the tMCAO+LV-OTULIN group (Fig. 3B(l), C) than in the tMCAO group (Fig. 3B(f)) or the tMCAO+LV-Scramble group (Fig. 3B(i)). In the contralateral cortex of all ischemic groups, Iba-1^+^ microglial cells existed diffusely in a resting shape, their Iba-1 immunoreactivity was quite weak, and activated-ramified or amoeboid Iba-1^+^ cells were rarely found at each time point after reperfusion (Fig. 3B(m–r)), the Iba-1 immunoreactivity in contralateral cortex of the Sham and tMCAO+LV-Scramble groups is not shown. Taken together, these results indicate that OTULIN overexpression limits the accumulation of microglia in the ischemic penumbra after cerebral ischemia, but this had no effect on the microglial cells in the contralateral cortex.

### OTULIN overexpression attenuated microglia activation in LPS-induced brain inflammation

**Fig. 4:**
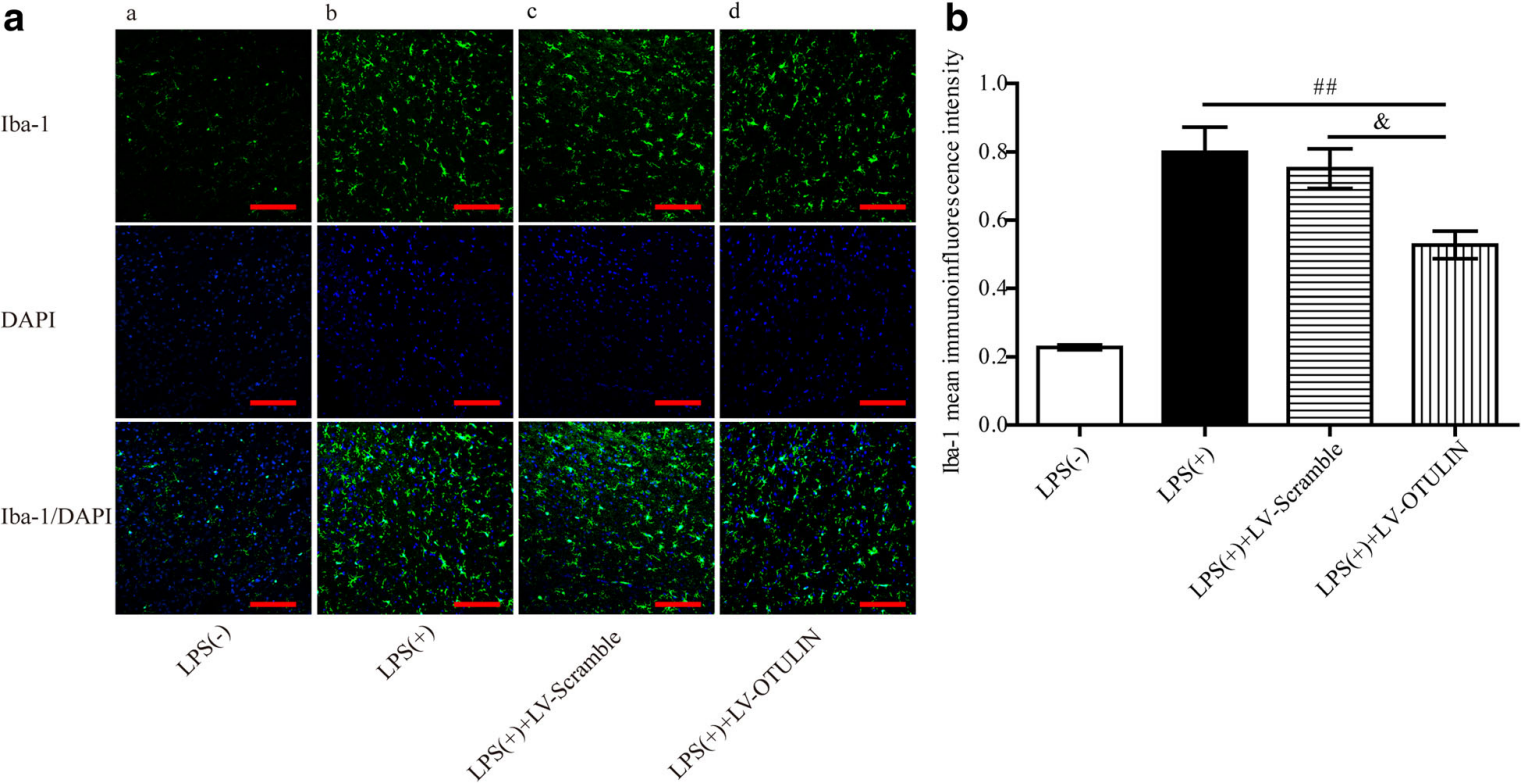
OTULIN overexpression decreased LPS-induced microglial activation. Pure brain inflammation without neuronal death was induced by IP injection of LPS, and tissues were collected 24 h later. **B** The mean Iba-1 immunofluorescence intensities in the **A** LPS(−) group (a), the LPS(+) group (b), the LPS(+)+LV-Scramble group (c), and the LPS (+)+LV-OTULIN group (d) were quantitatively analyzed (*n* = 6); scale bar = 75 μm. All data are presented as the means ± SEMs; ##*P* < 0.01 versus the LPS(+) group; &*P* < 0.05 versus the LPS(+)+LV-Scramble group

The extent of inflammation is often proportional to the infarct size [54, 56]. To exclude the possibility that the attenuation of microglia activation in OTULIN-overexpressing rats might be ascribed to the secondary effect of a reduction in infarct volume, we established a pure brain neuroinflammation rat model by IP injection of LPS, a direct promoter of microglial activation without a corresponding neuronal loss. The rats that received an injection of sterile saline served as the control group (LPS[−] group), and those that received the LPS injection served as the LPS(+) group. The rats were divided into four groups: the LPS(−) group, the LPS(+) group, the LPS(+)+LV-Scramble group, and the LPS(+)+LV-OTULIN group. The confocal immunofluorescence results showed that Iba-1^+^ microglial cells located in the cortex mainly existed in an activated-ramified state at 24 h after LPS treatment (Fig. 4A(b)), and the mean Iba-1 immunofluorescence intensity increased sharply compared with that in the LPS(−) group (Fig. 4A(a)). The quantitative analysis results (Fig. 4(b)) showed that overexpression of OTULIN (Fig. 4A(d)) resulted in an obvious decrease in mean Iba-1 immunofluorescence intensity compared to that in the LPS(+) (Fig. 4A(b)) and LPS(+)+LV-Scramble groups (Fig. 4A(c)).

### OTULIN was markedly increased in activated microglial cells

**Fig. 5:**
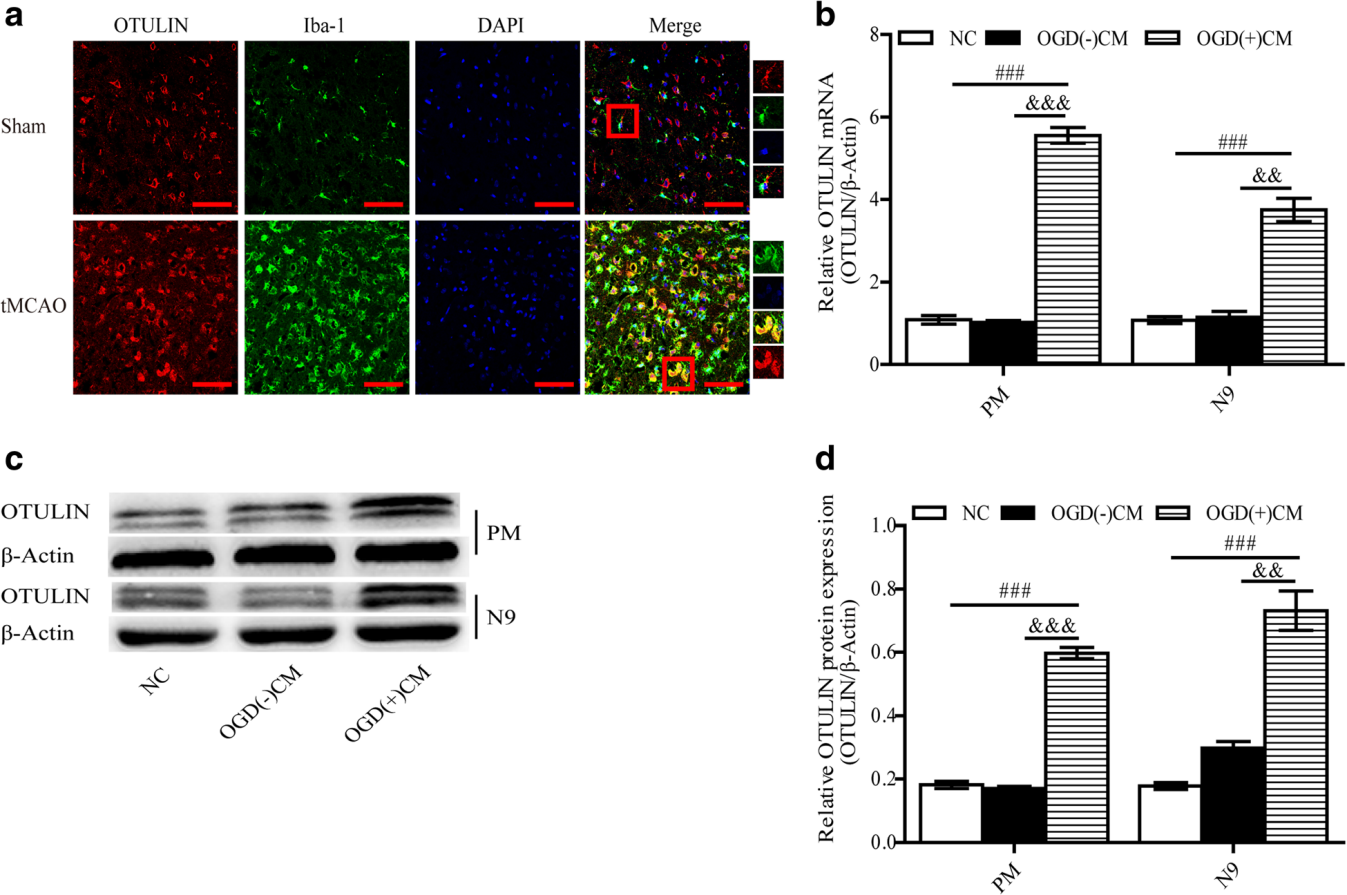
OTULIN was markedly increased in activated microglial cells. **a** Analysis of immunofluorescent confocal images showed that OTULIN was increased in activated microglial cells after ischemic stroke (OTULIN, red; Iba-1, green; DAPI, blue; scale bar = 75 μm; *n* = 6). The red boxes indicate enlarged areas. The levels of OTULIN mRNA (**b**, *n* = 6) and protein (**c**, **d**; *n* = 3) in PM and N9 cells were detected at 24 h after exposure to regular microglia medium, conditioned medium (CM) collected from OGD-treated neuronal cultures (OGD[+]CM), or OGD-untreated (OGD[−])CM. All values are presented as the means ± SEMs; ###*P* < 0.001 versus the NC group; &&&*P* < 0.001, &&*P* < 0.01 versus the OGD(−)CM group

To observe the expression of OTULIN in microglia after in vivo cerebral ischemia, a confocal immunofluorescence technique was performed to detect the double staining of OTULIN and Iba-1 in the tMCAO and Sham groups (*n* = 6 per group). Immunofluorescence results showed that Iba-1^+^ microglial cells were found in both hemispheres with a pronounced expression in the ischemic area at 72 h after reperfusion. The tMCAO group showed a greater increase in OTULIN immunoreactivity than did the Sham group. Interestingly, the expression of OTULIN was enriched in the activated microglial cells that possessed an amoeboid morphology, whereas little OTULIN was found in Iba-1^+^ resting microglia with a ramified shape in the Sham group (Fig. 5a).

We next quantitatively analyzed OTULIN expression levels in activated microglia in vitro. To mimic the in vivo stroke condition, we induced microglial cells with conditioned medium (CM) collected from OGD-treated neuronal cultures, which is a widely accepted cellular ischemic model [57, 58]. The groups were established as follows: the normal control (NC) group treated with regular microglial medium, the OGD(−)CM group cultured with OGD-untreated CM, and the OGD(+)CM group treated with CM that was not subjected to OGD. After 24 h of culture, the levels of OTULIN mRNA (Fig. 5b, *n* = 6 per group) and protein expression (Fig. 5c, d, *n* = 3 per group) were markedly increased in the PM and N9 cells in the OGD(+)CM group compared with the NC group or the OGD(−)CM group (Fig. 5b–d). Combined with the in vivo results, we could conclude that OTULIN levels were increased in ischemia-induced activated microglia.

### OTULIN overexpression depressed the expression of pro-inflammatory cytokines by modulating the NF-κB signaling pathway in focal ischemia/reperfusion rats

**Fig. 6:**
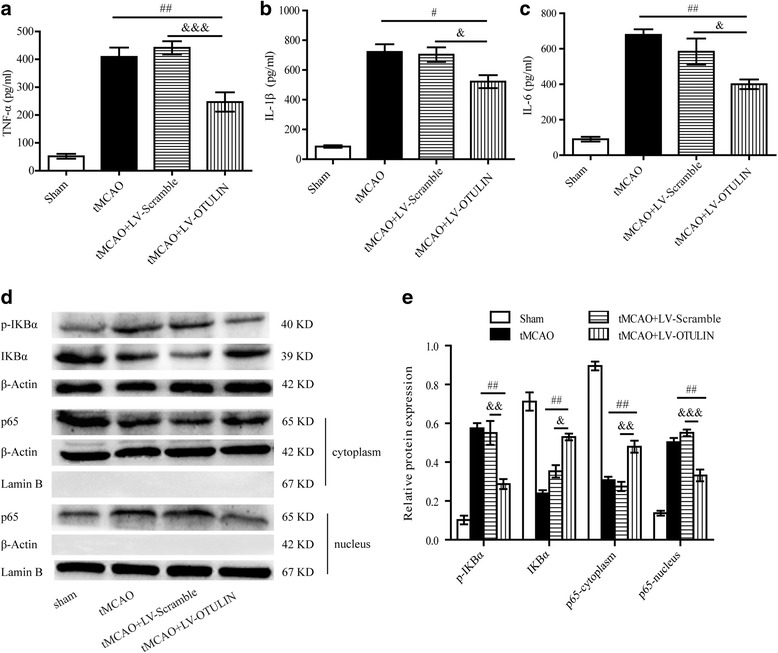
OTULIN overexpression reduced TNF-α, IL-1β, and IL-6 expression via the NF-κB signaling pathway at 24 h after reperfusion in tMCAO rats. The levels of TNF-α (**a**), IL-1β (**b**), and IL-6 (**c**) in the Sham, tMCAO, tMCAO+LV-Scramble, and tMCAO+LV-OTULIN groups were measured by ELISA (*n* = 6). **e** Quantitative analysis of the levels of p-IκBα and IκBα proteins in whole-cell preparations and p65 proteins in the cytoplasm and nucleus was detected by Western blot (**d**; *n* = 3). Data are presented as the means ± SEMs; ##*P* < 0.01, #*P* < 0.05 versus the tMCAO group; &&&*P* < 0.001, &&*P* < 0.01, &*P* < 0.05 versus the tMCAO+LV-Scramble group

To explore the effect of OTULIN on the production of inflammatory cytokines, ELISA was applied to evaluate the protein levels of TNF-α, IL-1β, and IL-6 at 24 h after cerebral ischemia. The rats were randomly divided into four groups: Sham group, tMCAO group, tMCAO+LV-Scramble group, and tMCAO+LV-OTULIN group (*n* = 6 per group for ELISA and *n* = 3 per group for Western blot). In line with the previous studies [7, 59, 60], ischemic stroke induced massive production of TNF-α, IL-1β, and IL-6. The treatment with LV-OTULIN in the tMCAO+LV-OTULIN group significantly decreased the contents of TNF-α (Fig. 6a), IL-1β (Fig. 6b), and IL-6 (Fig. 6c) compared to the levels of those proteins in the tMCAO group and the tMCAO+LV-Scramble group.

Next, we explored whether OTULIN overexpression decreased the expression of pro-inflammatory cytokines through the NF-κB pathway in tMCAO rats. Western blot was used to measure p-IκBα, IκBα, cytoplasmic p65, and nuclear p65 proteins in the ischemic area at 24 h after reperfusion. In the study, we detected enhanced degradation and phosphorylation of IκBα and nuclear translocation of NF-κB p65 in the tMCAO group compared to that in the Sham group (Fig. 6d, e). Moreover, OTULIN overexpression significantly attenuated NF-κB activity in the ischemic cortex, as determined by the inhibition of IκBα degradation and phosphorylation along with reduced nuclear translocation of p65, which manifested as less p65 in the nucleus and more p65 in the cytoplasm (Fig. 6d, e).

### OTULIN overexpression attenuated microglia-mediated neuroinflammation by modulating the NF-κB pathway in the in vitro ischemia condition

**Fig. 7:**
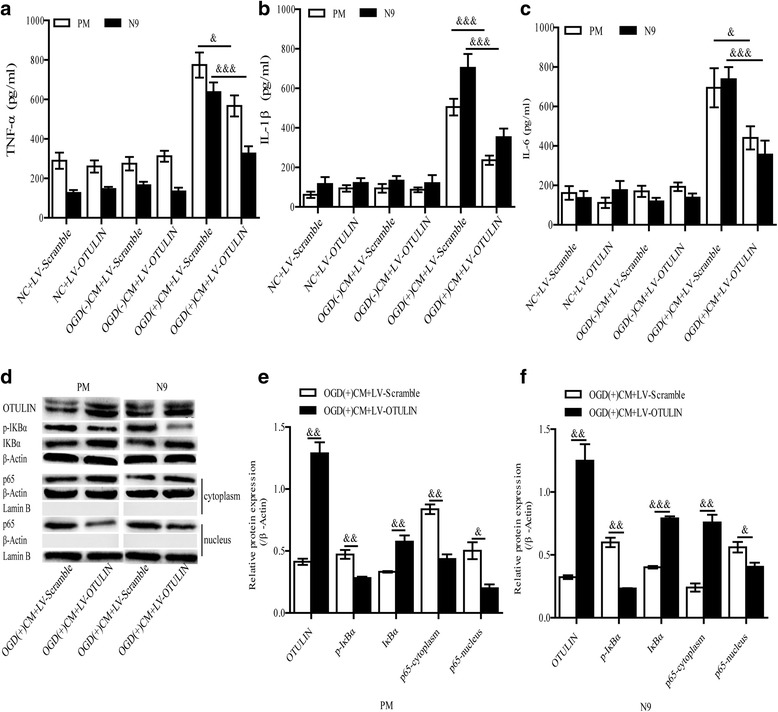
Overexpression of OTULIN suppressed the levels of the pro-inflammatory cytokines produced by activated PM and N9 cells through depressing the NF-κB pathway. ELISA was used to measure the levels of TNF-α (**a**; *n* = 6), IL-1β (**b**; *n* = 6), and IL-6 (**c**; *n* = 6) in the NC, OGD(−)CM, and OGD(+)CM groups of PM and N9 cells. Each group was transfected with the LV-OTULIN or LV-Scramble. OTULIN and NF-κB pathway-associated proteins including p-IκBα, IκBα, and cytoplasm/nucleus NF-κB p65 in PM (**d**, **e**; *n* = 3) and N9 (**d**, **f**; *n* = 3) cells were detected by Western blot and analyzed quantitatively. All values are presented as the means ± SEMs. ***P < 0.001, **P < 0.01, and *P < 0.05 versus the OGD(−)+LV-OTULIN group; &&&*P* < 0.001, &&*P* < 0.01, and &*P* < 0.05 versus the OGD(+)+LV-Scramble group

To further explore whether the post-ischemia induction of OTULIN acted directly via a microglial mechanism to modulate neuroinflammation, we activated microglial cells by using OGD(+)CM in vitro, which is a commonly used in vitro model for studying microglia-mediated neuroinflammation. PM or N9 cells transfected with LV-OTULIN or LV-Scramble were divided into NC, OGD(−)CM, and OGD(+)CM groups. ELISA results showed that only the OGD(+)CM markedly activated the PM and N9 cells, as evidenced by the robust production of TNF-α **(**Fig. 7a, *n* = 6 per group), IL-1β **(**Fig. 7b, *n* = 6 per group), and IL-6 **(**Fig. 7c, *n* = 6 per group), while the NC group and the OGD(−)CM group treated with LV-OTULIN or LV-Scramble displayed no changes in cytokine expression. The expression levels of these pro-inflammatory cytokines were significantly attenuated by LV-OTULIN but not by LV-Scramble (Fig. 7a–c).

Next, we analyzed whether OTULIN overexpression weakened the secretion of pro-inflammatory cytokines in PM and N9 cells via regulating the NF-κB pathway. Consistent with our in vivo results, we found that NF-κB activities induced by OGD(+)CM in the PM group **(**Fig. 7d, e, *n* **=** 3 per group), as well as in the N9 group **(**Fig. 7d, f, *n* **=** 3 per group), were significantly attenuated by LV-OTULIN, as shown by decreased IκBα degradation and phosphorylation and subsequently reduced nuclear translocation of p65. Collectively, these results reveal that OTULIN expression suppressed microglia-mediated neuroinflammation through the NF-κB pathway in the in vitro ischemic condition.

## Discussion

In-depth study of the pathological mechanisms of neuroinflammation induced by ischemic stroke is necessary for searching for novel therapy targets [61]. Microglia are regarded as the first responders [62] and the principal immune cells in the CNS that mediate neuroinflammation following cerebral ischemia [5, 63–65]. The NF-κB pathway is a key transcriptional pathway involved in stroke-induced neuroinflammation [15, 28, 66]. As a new member of the ovarian tumor (OTU) domain family, the deubiquitinase OTULIN was reported to be involved in acute systemic inflammation, chronic inflammation and autoimmunity via regulating the NF-κB pathway [40, 42]. Our previous studies demonstrated that other deubiquitinase, such as the zinc finger protein A20 and cylindromatosis (CYLD), functioned as negative regulators of NF-κB [67, 68] and were involved in regulating neuroinflammation through depressing neuronal NF-κB activity in ischemic stroke [69, 70]. In the present study, we found that OTULIN was enriched in activated microglia within the ischemic penumbra, while little OTULIN was detected in resting microglia, which suggests that OTULIN may function by regulating microglial cells. Interestingly, although OTULIN, A20, and CYLD remove specific ubiquitin chains on IKK complexes, the deubiquitination activity of OTULIN appears to be more critical than A20 and CYLD [39, 41]. Hence, it was logical and valuable for us to assume that OTULIN played a protective role against ischemic stroke by modulating neuroinflammation.

Initially, we examined the time course of OTULIN expression in the early stage of ischemic stroke. The present study revealed that low levels of OTULIN mRNA and protein expression were detected in the Sham rats, which indicates that OTULIN is constitutively expressed in the brain. The endogenous OTULIN expression was rapidly and persistently increased in the ischemic penumbra of the cerebral cortex within 72 h following ischemic stroke, which indicates that rats may resist ischemic brain injury by mobilizing endogenous OTULIN. Due to the potent anti-inflammatory effect of OTULIN, we utilized gene interference to enhance OTULIN expression and explored the role and mechanism of OTULIN in ameliorating cerebral ischemic injury. As expected, OTULIN overexpression obviously alleviated ischemia-induced early brain damage as evidenced by reduced infarct volume, improved neurological function, and reduced neuronal loss in the ischemic penumbra. Therefore, OTULIN could be a potential endogenous protective regulator against ischemic brain injury.

OTULIN is expressed in peripheral immune cells including lymphocytes, natural killer (NK) cells, and prominently in dendritic cells and macrophages. Targeted deletion of OTULIN in myeloid cells caused chronic inflammation and autoimmunity by amplifying NF-κB activity [40]. However, its expression in microglial cells was completely unknown. As post-ischemic microglia proliferation peaks at 48 to 72 h and lasts for several weeks [5, 60], we chose to observe OTULIN expression in microglia at 72 h. Using an immunofluorescence approach, we observed that cerebral ischemia-induced OTULIN expression in activated microglia with an amoeboid shape, whereas OTULIN expression was low in the ramified microglia. Moreover, in vitro OTULIN levels were significantly upregulated in the PM and N9 microglial cells stimulated with OGD(+)CM. These data suggest that this upregulation of OTULIN expression may be a potential mechanism of resistance to microglial activation. However, OTULIN was not necessarily involved in the inflammatory responses in the immune cells that contained OTULIN. Damgaard et al. [40] reported that mice with an OTULIN deficiency in lymphocytes did not manifest overt acute inflammatory phenotypes, whereas deletion of OTULIN in myeloid cells generated an obvious inflammation phenotype and spontaneous NF-κB activation, which suggests a crucial and cell-specific effect of OTULIN in maintaining immune homeostasis [41]. Consistent with former studies [5, 56, 59, 60], the current study also detected a rapid and time-dependent activation of microglia following cerebral ischemia, along with a gradual increase in amoeboid microglia in the ischemic penumbra, while microglial cells remained at a basal level in the contralateral cortex with a ramified state. OTULIN overexpression significantly suppressed the activation of Iba-1^+^ microglial cells located in the cortical penumbra. To exclude the possibility that the effect of OTULIN on reducing microglia activation is an indirect outcome of its neuroprotective role [56], a commonly used LPS-induced pure brain inflammation model without brain cell death served as a positive control. Importantly, we observed a similar effect: OTULIN overexpression could obviously inhibit the activation of microglia in the LPS-induced inflammation model. Collectively, these data suggest that OTULIN plays a direct role in the activation of microglial cells in focal cerebral ischemia/reperfusion rats.

In the acute phase of cerebral ischemia, the release of various pro-inflammatory cytokines that are produced by activated microglia is the main mechanism that leads to ischemic inflammatory injury, and the NF-κB pathway is the critical signal transduction pathway that mediates this process [71, 72]. Consistent with the finding that OTULIN overexpression was shown to delay NF-κB activation of TNF, whereas its knockdown was reported to increase the output of NF-κB signaling [36, 38, 39], we found that OTULIN overexpression strongly depressed NF-κB activity and reduced the release of TNF-α, IL-1β, and IL-6, which are the primary pro-inflammatory cytokines that aggravate brain injury. To some extent, our study indicates that OTULIN exerts an anti-inflammatory effect by modulating microglia-mediated neuroinflammation. More importantly, we next revealed a direct mechanism of microglial cells underlying the anti-neuroinflammation effects of OTULIN in the focal cerebral ischemia/reperfusion rats. The CM from the OGD(+) neuronal culture was used to induce PM and N9 cells, which is a classic way to mimic an in vivo ischemic stroke [57]. Our results showed that PM and N9 microglial cells were obviously activated at 24 h after stimulation with OGD(+)CM, as shown by significant increases in TNF-α, IL-1β, and IL-6. OTULIN overexpression significantly suppressed the production of TNF-α, IL-1β, and IL-6 in PM and N9 cells via inhibiting the nuclear translocation of NF-κB p65 but had no effect on the pro-inflammatory cytokines secreted by un-activated microglia, which directly indicates that OTULIN suppressed microglia-mediated neuroinflammation by inhibiting the NF-κB pathway and attenuated the release of inflammatory mediators under the in vitro ischemic condition.

**Fig. 8:**
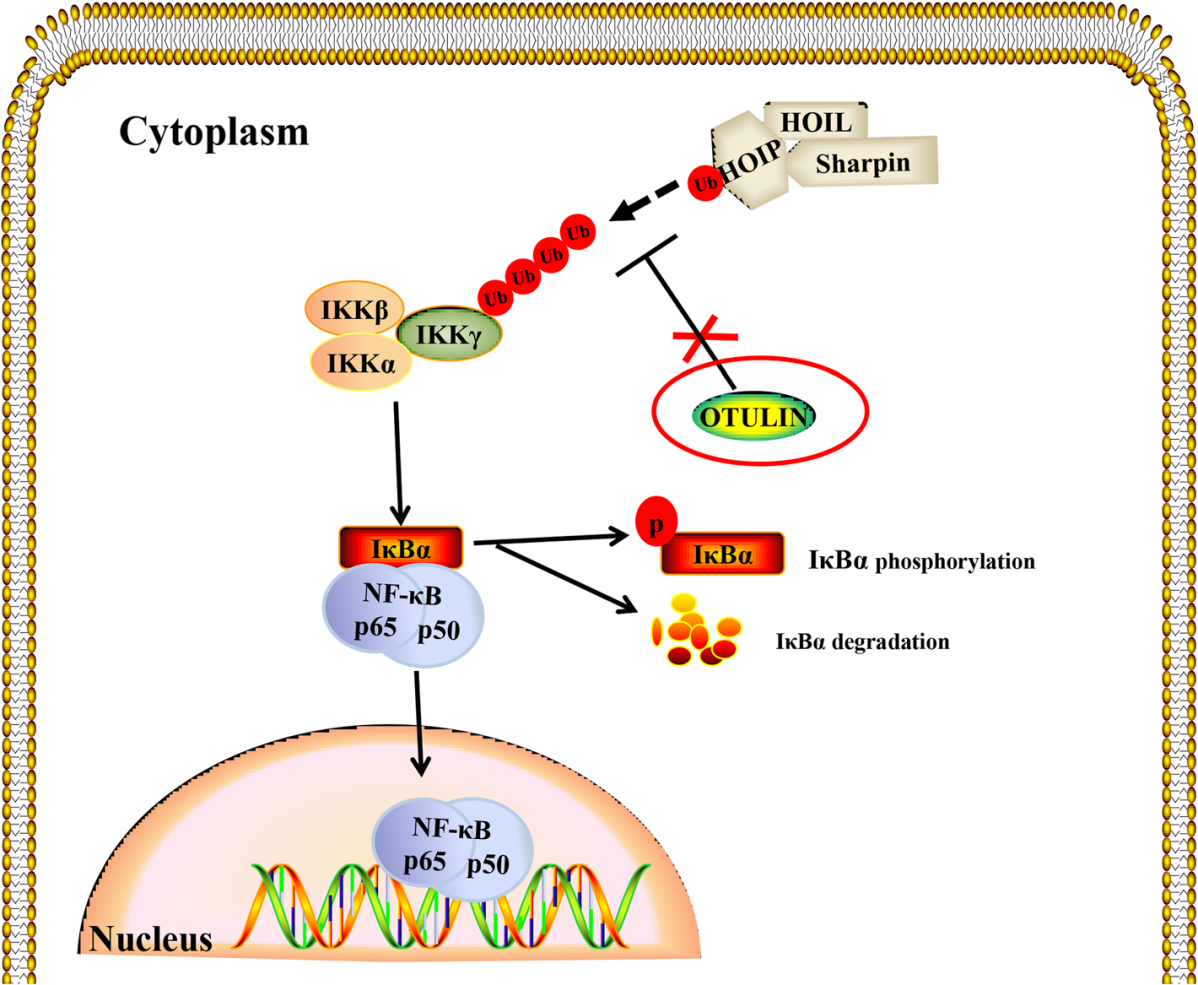
Schematic representation of OTULIN regulating the NF-κB pathway in ischemic stroke. Ischemic stroke induced rapid activation of the NF-κB pathway, mainly shown by the activation of IKKs, the degradation and phosphorylation of IκBα, and the increased nuclear translocation of NF-κB p65. The activated NF-κB pathway leads to microglial activation along with a sharp increase in the production of pro-inflammatory cytokines and the occurrence of inflammatory responses in the brain. LUBAC, which consists of HOIP, HOIL-1, and SHARPIN, participates in the assembly of ubiquitin chains on IKK complexes. The deubiquitinase OTULIN interacts with HOIP to cleave the Met-1 Ub on IKKγ, thus resulting in depression of the NF-κB pathway. OTULIN overexpression obviously inhibited the excessive activation of microglia and the production of inflammatory responses through suppressing the phosphorylation and degradation of IκBα and subsequently attenuating the nuclear translocation of NF-κB p65

Here, for the first time, we have revealed that the overexpression of OTULIN ameliorated microglial cell activation and neuroinflammation by repressing the NF-κB pathway in cerebral ischemia/reperfusion rats. To some extent, our study fills the gap in OTULIN research in the CNS and presents the new idea that OTULIN may function through regulating microglia-mediated neuroinflammation as shown in Fig. 8. However, it may not be the only mechanism by which OTULIN exerts a neuroprotective role following cerebral ischemic insult. It has been proven that appropriate suppression of neuronal NF-κB activity can have a neuroprotective effect in the early stage of ischemic stroke [70, 73]. That inspired us to consider whether the neuroprotective role of OTULIN might be achieved through the attenuation of microglia-mediated neuroinflammation alone or along with neuronal mechanisms, a possibility that needs further investigation.

Ubiquitination is a crucial posttranslational modification that regulates various processes. OTULIN deficiency can cause amplification of the Met-1 Ub-regulated canonical NF-κB pathway. How the overexpression of OTULIN attenuated NF-κB activation at the ubiquitination level was not explored in the current study. The linear ubiquitin chain assembly complex (LUBAC), composed of HOIP, HOIL-1, and SHARPIN, is critical in the assembly of ubiquitin chains on IKKγ. OTULIN interacts with the N-terminal PUB domain of HOIP via an evolutionarily conserved PUB-interacting motif to remove the Met-1 Ub on IKKγ [37–39], thus leading to the inactivation of IKK. Inactivation of IKK in turn attenuates NF-κB activity. Therefore, there are many issues that remain to be elaborated, some of which could be addressed via the following questions. Does OTULIN overexpression affect its association with HOIP? Can OTULIN overexpression reduce Met-1 Ub levels linked to IKKγ? Does OTULIN overexpression influence the other components of the LUBAC, such as HOIL and SHARPIN?

## Conclusions

n summary, this study is the first to demonstrate that overexpression of OTULIN in an ischemic stroke model exerted a neuroprotective role via reduced infarct, improved neurological function deficits, and less neuronal loss in focal cerebral ischemia/reperfusion rats. Moreover, OTULIN overexpression inhibited the excessive activation of microglial cells and the production of pro-inflammatory mediators through depressing NF-κB signaling pathways in vivo and in vitro. Although further studies on the precise mechanism remain to be explored, we present the novel idea that OTULIN could be a promising candidate for the treatment of ischemic stroke.

## Additional file

Additional file 1: **Figure S1.** The expression of OTULIN before inducing tMCAO was significantly enhanced via gene interference by LV-OTULIN. The rats were randomly divided into three groups: normal control (NC), normal control receiving LV-Scramble (NC+LV-Scramble), and normal control receiving LV-OTULIN (NC+LV-OTULIN). (A) The expression of OTULIN mRNA in each group was detected by RT-qPCR (*n* = 5). (B) The expression of OTULIN protein in each group was examined by Western blotting. (C) The histogram shows the quantitation of OTULIN protein levels (*n* = 3). All values are presented as the means ± SEMs; @@@*P* < 0.05 versus the NC group; $$$P < 0.05 versus the NC+LV-Scramble group. (TIFF 9863 kb)

## Abbreviations

CNS: Central nervous system
ELISA: Enzyme-linked immunosorbent assay
Iba-1: Ionized calcium-binding adapter molecule 1
IL-1β: Interleukin-1 beta
IL-6: Interleukin-6
IP: Intraperitoneal
IκBα: NF-κB inhibitor protein alpha
NF-κB: Nuclear transcription factor kappa B
RT-qPCR: Real-time quantitative polymerase chain reaction
tMCAO: transient Middle cerebral artery occlusion
TNF-α: Tumor necrosis factor alpha
TTC: 2,3,5-Triphenyltetrazolium chloride
