# Supplementary figures and images for: Lentivirus-mediated overexpression of OTULIN ameliorates microglia activation and neuroinflammation by depressing the activation of the NF-κB signaling pathway in cerebral ischemia/reperfusion rats
Source: J Neuroinflammation. 2018 Mar 15;15:83. doi: 10.1186/s12974-018-1117-5 (PMC5856386; doi:10.1186/s12974-018-1117-5)

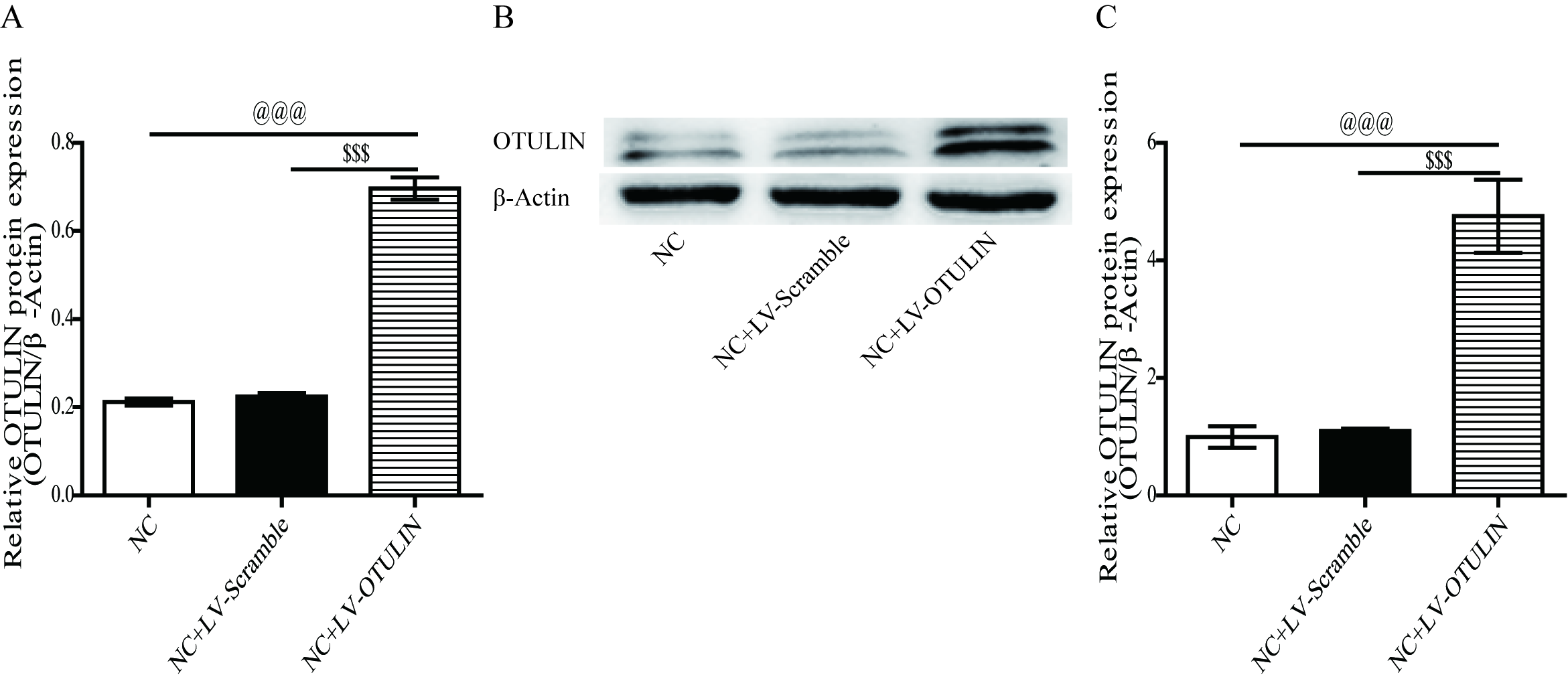

Supplement: Supplementary file 1 — Figure S1. The expression of OTULIN before inducing tMCAO was significantly enhanced via gene interference by LV-OTULIN. The rats were randomly divided into three groups: normal control (NC), normal control receiving LV-Scramble (NC+LV-Scramble), and normal control receiving LV-OTULIN (NC+LV-OTULIN). (A) The expression of OTULIN mRNA in each group was detected by RT-qPCR (n = 5). (B) The expression of OTULIN protein in each group was examined by Western blotting. (C) The histogram shows the quantitation of OTULIN protein levels (n = 3). All values are presented as the means ± SEMs; @@@P < 0.05 versus the NC group; $$$P < 0.05 versus the NC+LV-Scramble group. (TIFF 9863 kb) [file 12974_2018_1117_MOESM1_ESM.tif]
